# Healthcare and economic burden of ANCA-associated vasculitis in Italy: an integrated analysis from clinical and administrative databases

**DOI:** 10.1007/s11739-020-02431-y

**Published:** 2020-07-14

**Authors:** Luca Quartuccio, Elena Treppo, Francesca Valent, Salvatore De Vita

**Affiliations:** 1 Department of Medicine (DAME), Clinic of Rheumatology, Department of Medicine (DAME), ASU FC, University of Udine, Udine, Italy; 2 Igiene Ed Epidemiologia Clinica, ASU FC, Udine, Italy

**Keywords:** Vasculitis, ANCA, Autoantibody, Cost, Healthcare, Hospitalization, Granulomatosis, Polyangiitis

## Abstract

ANCA-associated vasculitides (AAV) comprise a group of systemic vasculitides characterized by inflammation of small-sized blood vessels leading to multi-organ involvement. The worldwide annual incidence of AAV ranges from 1.2 to 3.3 cases per 100 000 individuals with a prevalence of 4.6–42.1 cases per 100 000 individuals. The prevalence of AAV is geographically heterogeneous; therefore, regional epidemiological studies can be more informative to improve health care systems. Even though clinicians are aware that the healthcare burden and the risk of hospitalization of AAV appear high, data on hospitalization and cost of illness due to AAV are still scarce or even lacking. This study aims to characterize the economic burden of AAV in Friuli Venezia Giulia (FVG), Italy. Thus, a retrospective study was conducted through the integration of many administrative health databases of the FVG as the source of information. From data integration, we estimated that more than two-thirds of AAV patients showed at least one hospitalization in their medical history, most frequently caused by the disease itself or superimposed infections. Around 10% of patients developed end-stage renal disease. In an 8-year follow-up, the overall healthcare cost was € 1,215,078, corresponding to € 6,168 patient-year. ANCA-positive patients showed much higher costs than ANCA-negative patients did. Overall, AAV are rare diseases, but imply very high healthcare costs. Early diagnosis and optimal treatment probably still remain unmet needs for AAV.

## Introduction

ANCA-associated vasculitides (AAV) are a group of systemic vasculitides characterized by inflammation of small-sized blood vessels leading to multi-organ involvement [1–5]. AAV include three major clinic-pathologic entities: granulomatosis with polyangiitis (GPA), microscopic polyangiitis (MPA), and eosinophilic granulomatosis with polyangiitis (EGPA) [6, 7]. AAV are rare diseases, with a worldwide annual incidence ranging from 1.2 to 3.3 cases per 100 000 individuals with a prevalence of 4.6–42.1 cases per 100 000 individuals [2]. Nevertheless, the incidence and prevalence of AAV have been progressively increasing in the last decades [2]. Among the subsets of AAV important geographic differences have been observed [2, 8]. In Europe and Australia, a higher proportion of GPA versus MPA has been observed [2, 9]. Differently, in Asia (especially Japan), a higher proportion of MPA versus GPA has been observed [2]. The improvement in induction therapy has changed acute and life-threatening diseases to chronic ones associated with relapses, organ damage accumulation, and long-term immunosuppressive treatment side effects [2, 10–16]. Therefore, AAV shows a high healthcare burden with a high risk of hospitalization. However, data on hospitalization and the cost of illness due to AAV are still limited [3, 17]. Herein, we report the healthcare burden and the direct costs of the illness of AAV in Friuli Venezia Giulia (FVG), a region in the northeast of Italy. Importantly, two different data sources were used and compared, i.e., administrative healthcare databases and clinical databases. Second, the costs of illness from a European healthcare system are information currently lacking for AAV in the literature.

## Methods

The Regional Health Information System of FVG (about 1 200 000 inhabitants) and clinical electronic chart records from our Hospital Clinic were used as the source of information for this retrospective cohort study.

The system covers the entire regional population and includes various electronic health administrative databases that can be linked with one another on an individual basis through a unique encrypted ID identifier. The database of the regional potential health care beneficiaries (including demographic information and the residential history of all of the subjects living in the region), the hospital discharge database, the pharmaceutical prescription database, and the database of exemptions from medical charges were used for this study.

The hospital discharge database includes records from all of the regional hospitals (both public and private accredited to the public health system) and those regarding admissions of regional residents to extra-regional hospitals. The pharmaceutical prescription database contains information on all of the medications prescribed by the physicians working in the public health system except those paid out-of-pocket. The database of exemptions from medical charges includes records on all of the potential health care beneficiaries who are entitled, because of low income, age, or chronic diseases, to receive free medications and outpatient specialist care. Notably, the Emergency Department (ED) database is accessible, but, contrary to the procedures for hospital admissions or outpatient ambulatory care, the ED has no standard tariff associated with healthcare episodes. Thus, we have access to ED records, but they do not include any cost information.

The Italian Ministry of Health assigns codes to all of the diseases that entitle patients to exemptions. Currently, they include approximately 100 chronic and disabling diseases including the groups of rare diseases [18], where GPA, MPA, and EGPA are identified with the following codes: RG0070, RG0020, and RG0050, respectively. In the Italian healthcare system, only the specialists have the authorization to provide the exemption codes for rare diseases, and since the year 2010, a great effort has been made in our Region to keep the rare disease registry up-to-date, with the aim of filling such gap.

The cohort included all of the subjects living in FVG who received an exemption from medical charges because of a diagnosis of either GPA, MPA, or EGPA, according to the corresponding exemption code from 2010 to 2018. The subjects were observed from the date of first release of the exemption and followed until they moved outside the region, died, the outcome of interest occurred, or December 31, 2018, whichever came first. The outcome of interest was the event of hospitalization or death. Of note, we restricted the time frame of observation to the years from 2013 to 2018 as concerns the analysis on the whole regional population, i.e., starting from the year in which the laboratory analyses were centralized in the Laboratory of the Hospital of Udine, in particular for the autoimmunity tests, while we analyzed a longer time frame (2010–2018) when considering the province of Udine (about 530 000 inhabitants, the half of the whole regional population). For the best comparison, regarding the local retrospective cohort from our Clinic, we included in the analysis all the patients examined in Rheumatology Clinic of Udine (Referral Centre for AAV in FVG) who received diagnosis of GPA, MPA, and EGPA from 2010 to 2018.

### Statistical analysis

The frequency distribution of the baseline cohort characteristics and events of interest was calculated. The statistical significance of differences in the variable distribution between patients who experienced the event of interest and the others was assessed using the Chi-squared test for categorical variables, the t test for continuous variables with normal distribution, and Wilcoxon’s rank-sum test for continuous variables with non-normal distribution. Normality was assessed using the Kolmogorov–Smirnov test.

Kaplan–Meier curves were calculated to describe the event-free survival of patients, both overall and by treatment groups. The log-rank test and Wilcoxon’s test were used to assess the significance of differences in survival. *P* < 0.05 was considered statistically significant.

All of the analyses were assessed using SAS v9.4 (SAS Institute Inc., Cary, NC, USA.).

### Compliance with ethical standards

The authors assert that all of the procedures contributing to this work comply with the ethical standards of the relevant national and institutional committees on human experimentation and the Helsinki Declaration of 1975 as revised in 2008. This article does not contain any studies of human or animal subjects performed by any of the authors. Since this analysis was based on anonymous administrative data, patient informed consent and Ethical Committee approval were not required in Italy. As concerns the analysis that was conducted on electronic clinical chart records, patients admitted to our Hospital were asked to sign an informed consent for using their own data for research purpose.

## Results

### AAV epidemiology by integrated analysis of the administrative databases

**Fig. 1:**
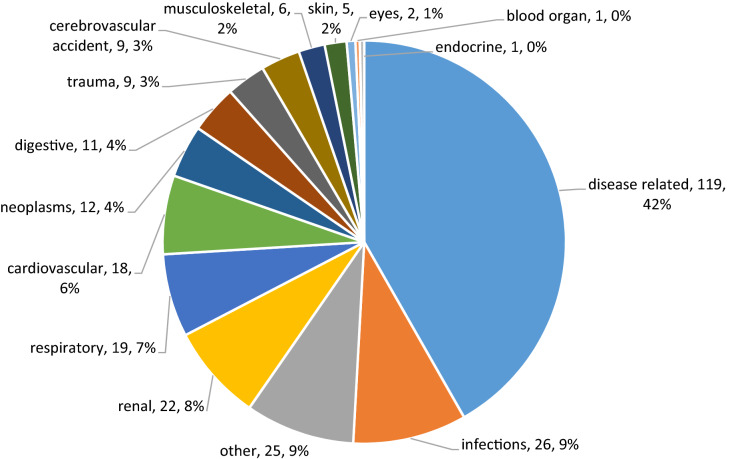
Distribution of the categories of main discharge diagnosis for 285 hospitalizations of patients with AAV in FVG (years 2013–2018). the following ICD-9-CM codes were considered “disease related”: 287.0 “Allergic purpura”, 288.3 “Eosinophilia”, 381.4 “Nonsuppurative otitis media, not specified as acute or chronic”, 423.0 “Hemopericardium “, 437.4 “Cerebral arteritis”, 446.4 “Wegener's granulomatosis”, 447.6 “Arteritis, unspecified”, 473.0 “Chronic maxillary sinusitis”, 473.2 “Chronic ethmoidal sinusitis”, 518.3 “Pulmonary eosinophilia”, 580.4 “Acute glomerulonephritis with lesion of rapidly progressive glomerulonephritis”, 582.4 “Chronic nephritis with lesion of necrotizing glomerulitis”, 582.9 “Chronic glomerulonephritis with unspecified pathological lesion in kidney”, 584.5 “Renal failure with (acute) tubular necrosis”, 375.53 “Stenosis of lacrimal canaliculi”, 375.56 “Stenosis of nasolacrimal duct, acquired”, 381.10 “Chronic serous otitis media, simple or unspecified”, 420.90 “Acute pericarditis, unspecified”, 446.29 “Other specified hypersensitivity angiitis”, 478.74 “Stenosis of larynx”, 519.19 “Unspecified disease of respiratory system”, 582.89 “Chronic glomerulonephritis with lesion of: exudative nephritis”

Between the years 2013 and 2018, 103 patients with diagnostic code for AAV were identified in FVG, accounting for 8.58 cases/100 000 inhabitants (Table 1). Among these, patients with at least one hospitalization or death were 74/103 (71.8%). 7/103 (6.8%) died during the observation period. The whole number of hospitalizations was 285 in 74 patients (rate of 57 events per year), with an inpatient prevalence of 23.2 cases per 100 000 admissions. Fifty-five out of 74 (74.3%) patients experienced more than one hospitalization. In most cases, the cause of hospitalization was a condition with high probability of being secondary to vasculitis (119/285, 41.8%) or the disease itself in more than half of cases (62/119, 52.1%, ICD-9-CM code 446.4, “Wegener’s granulomatosis”) (Fig. 1). Infections were the second cause of hospitalization (26/285, 9.1%) (Fig. 1). In 10/103 patients (9.7%), end-stage renal disease (ESRD) was recorded as an event. The presence of at least one positivity for ANCA antibodies was documented in 76/103 patients (73.8%), mainly in patients carrying GPA. Globally, ANCA positivity tended to be associated with a greater likelihood of an event (hospitalization/death) (HR 1.75, 95% CI 0.97–3.16, *p* = 0.06, Log-Rank test), irrespectively from disease diagnosis and after correction for age and gender. The first event occurred in 50% of ANCA-positive patients within 180 days (95% CI 84–297) from the diagnosis, while in 50% of ANCA-negative patients in 859 days (95% CI 87–1695). Notably, six out of the seven deaths occurred in ANCA-positive patients.

**Table 1:** Comparison between Regional Health Information System of Friuli Venezia Giulia and clinical electronic chart records from our Hospital Clinic

AAV patients	Administrative healthcare databases					Clinical databases
	Residents in Friuli Venezia Giulia	Residents in the province of Udine				Rheumatology Clinic of Udine
Period of observation	2013–2018	2010–2018				2010–2018
	Mean (SD) or *N* (%)					
N. Pts. AAV	103	57				58
Disease diagnosis (GPA; EGPA; MPA)	40 (38.8%); 39 (37.9%); 24 (23.3%)	20 (35.1%); 21 (36.8%); 16 (28.1%)				30 (51.7%); 19 (32.8%); 9 (15.5%)
Mean age	55.1 (16.2)	54.5 (17.5)				57.8 (14.3)
ANCA positivity	76/103 (73.8%)	44/57 (77.2%)				47/58 (81)%
N. hospitalizations	285	126				82
Hospitalization rate (per year)	57	–				10.3
Pts. with at least one event (hospitalization or death)	74/103 (71.8%)	–				47/58 (81%)
Pts. with ≥ 2 hospitalizations	55/74 (74.3%)	–				18/47 (38.3%)
Hospitalization for disease itself	119/285 (41.8%)	60/126 (47.6%)				56/82 (68.3%)
Hospitalization for infective disease	26/285 (9.1%)	–				9/82 (10.9%)
ESRD	10/103 (9.7%)	–				5/58 (8.6%)
Death	7/103 (6.8%)	4/57 (7%)				6/58 (10.3%)
Mortality rate (per year)	1.4	0.5				0.75
		GPA	EGPA		MPA	
Estimated cost per patient-year (€)		5199	2329		4771	
		ANCA +		ANCA-		
Estimated cost per patient-year (€)		7058		2559		

*AAV* ANCA-associated vasculitis, *ANCA* anti-neutrophil cytoplasmic antibodies, *ESRD* end-stage renal disease

**Fig. 2:**
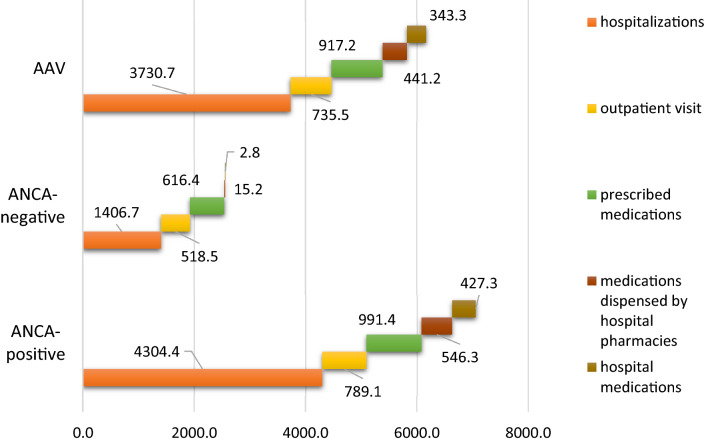
The overall estimated healthcare costs (€) per person-year for 57 AAV patients, residents in the province of Udine from 2010 to 2018, divided into ANCA-positive and ANCA-negative

**Fig. 3:**
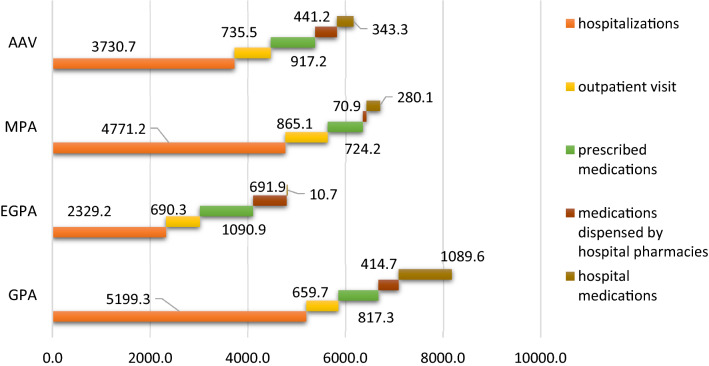
The overall estimated healthcare costs (€) per person-year for 57 AAV patients, residents in the province of Udine from 2010 to 2018, also divided into each entity (GPA, MPA, and EGPA)

For the province of Udine, 530 000 inhabitants served by the University Hospital of Udine and by the reference third-level center for rare rheumatologic diseases; laboratory results have been available since 2010. Thus, we evaluated the cost of illness in patients suffering from AAV and residents in the province of Udine from 2010 to 2018. In the province, from 2010 to 2018, 57 patients (201 patient-years) with AAV were identified. GPA, EGPA, and MPA were diagnosed in 20 (35.1%), 21 (36.8%), and 16 (28.1%) patients, respectively. They were ANCA-positive in 44/57 (77.2%); of which GPA, EGPA, and MPA were diagnosed in 18 (40.9%), 15 (34.1%), and 11 (25%) patients, respectively. The mean age at diagnosis was 54.5 (17.5) years. Again, the disease itself was the main cause of hospitalization in almost half of the hospital discharges (60/126, 47.6%). Four patients died during the observation period due to vasculitis itself (1/4), pneumonia (2/4), or haematological malignancy (1/4). Time to the first event (hospitalization or death) was significantly lower in ANCA-positive AAV patients than in ANCA-negative AAV patients, ANCA-positive AAV patients showing a three times higher risk than ANCA-negative patients (HR 3.38 95% CI 1.13–10.18, *p* = 0.03). Total estimated cost was € 1,215,078, corresponding to € 6,168 per patient-year. Costs for ANCA-positive AAV patients were much higher than for ANCA-negative patients (€ 1,115,253 vs € 99,825, and € 7,058 per person-year vs € 2,559 per person-year, respectively) (Fig. 2). GPA and MPA showed the highest costs if compared to EGPA [GPA: € 239,168 (€ 5,199 per person-year) vs MPA: € 281,502 (€ 4,771 per person-year) vs EGPA: € 214,287 (2,329 per person-year), respectively] (Fig. 3). Interestingly, hospital medications and medications dispensed by hospital pharmacies for EGPA patients represent a minor burden of expenditure as compared to MPA and GPA, whereas costs for prescribed medications are higher for EGPA patients (Fig. 3). Finally, costs for hospitalization were the highest among the direct costs of illness [€ 734,957 (€ 3,731 per person-year) vs other costs € 480,121 (€ 2,437 per person-year)].

### AAV epidemiology based on local clinical electronic chart records

**Fig. 4:**
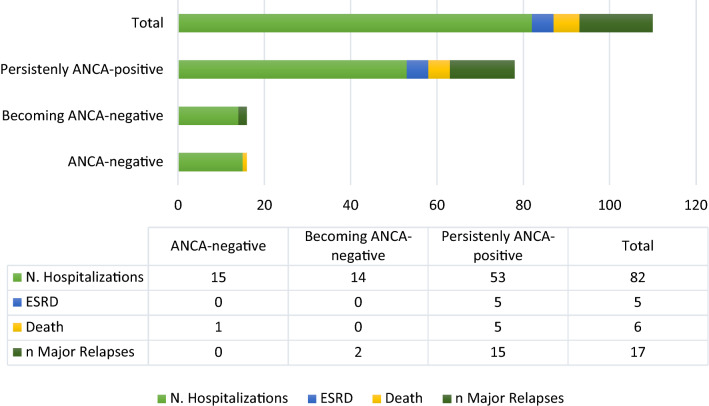
Numbers of events in the 58 AAV patients followed by the Rheumatology Clinic of Udine (2010–2018). The patients have been divided into three categories: patients persistently ANCA-positive, patients becoming ANCA-negative, and patients ANCA-negative

Further detailed information on patients with AAV was obtained using data from the Rheumatology Clinic of Udine, Referral Centre for AAV in FVG. Using our database, 58 patients (34 females, 24 males) with AAV diagnosis from 2010 to 2018 were identified. The mean age at the diagnosis of disease was 57.8 (14.3) years. Patients’ diagnosis included GPA in 30 cases (51.7%), EGPA in 19 cases (32.8%), and MPA in 9 cases (15.5%). They were ANCA-positive in 47/58 (81%), in particular 24/47 (51.1%) cANCA/PR3 and 23/47 (48.9%) pANCA/MPO. The main characteristics of the patients are summarized in Table 2. Patients with at least one hospitalization were 47/58 (81%). Eighteen out of 47 (38.3%) patients experienced more than one hospitalization. The whole number of hospitalizations was 82 in 47 patients (10.3 events per year). The disease itself was the main cause of hospitalization in more than half of the hospital discharges [56/82 (68.3%)]; infections were the second one [9/82 (10.9%)]. 5/58 (8.6%) patients developed ESRD and 6/58 (10.3%) patients died during the observation period. ANCA positivity seems to be associated with a greater likelihood of an event, i.e., hospitalization [67/82 (81.7%)] and death [5/6 (83.3%)]. Interestingly, patients becoming ANCA-negative showed a better outcome than persistently ANCA-positive patients did (Fig. 4).

**Table 2:** Characteristics of 58 AAV patients followed by the Rheumatology Clinic of Udine (2010–2018)

Demography	
Age, years	57.8 ± 14.3
Female gender	34 (58.6%)
AAV diagnosis	
GPA	30 (51.7%)
EGPA	19 (32.8%)
MPA	9 (15.5%)
BVAS v3	13.9 ± 6.7
Baseline vasculitis involvement	
Constitutional symptoms	47 (81%)
ENT	35 (60.3%)
Lungs	29 (50%)
Kidney	19 (32.8%)
Nervous system	19 (32.8%)
Skin	19 (32.8%)
Cardiovascular	6 (10.3%)
Gastrointestinal	6 (10.3%)
Eye	2 (3.4%)
ANCA status	
Positive ANCA	47 (81%)
cANCA/PR3	24 (51.1%)
pANCA/MPO	23 (48.9%)

*AAV* ANCA-associated vasculitis, *GPA* granulomatosis with polyangiitis, *EGPA* eosinophilic granulomatosis with polyangiitis, *MPA* micropolyangiitis, *BVAS* Birmingham Vasculitis Activity Score, *ENT* ear-nose-throat involvement, *ANCA* anti-neutrophil cytoplasmic antibodies, *PR3* proteinase 3, *MPO* myeloperoxidase. Variables are reported as mean ± SD or *N* (%)

## Discussion

In the present study, we performed a global assessment of inpatient epidemiology and the economic burden of AAV in FVG (around 1 200 000 people) by integrating information coming from different sources, i.e., administrative healthcare databases and clinical databases (Table 1). This comparative analysis should be relevant for highlighting strengths and limitations of each source of data, and for addressing future researches in this field. Notably, cost estimation reported by us represents the first data available in the literature coming from a European healthcare system.

The management of AAV has evolved considerably in the recent decades, allowing improvements in outcomes and overall survival [19–22]. Nevertheless, the disease itself and, importantly, superimposed infections are still the main causes of hospitalization in AAV [3, 23]. In addition, still in the last decade, almost 10% of patients develop ESRD during the follow-up. Both the methodological approaches of our study led to this observation. Thus, the unmet needs of early diagnosis, in particular for renal involvement, and novel treatments, especially carrying significant steroid-sparing effect, clearly emerged from these results [24, 25]. Second, the ANCA status was confirmed to be an important prognostic factor [14, 26]. ANCA-positive patients, in particular ANCA/PR3-positive patients, more frequently show a relapsing–remitting disease [14]. Mortality and hospitalization rate were both significantly related to the presence of ANCA in our study. In addition, ANCA-positive patients showed a much shorter time to the first hospitalization than ANCA-negative patients, thus implying a more aggressive systemic disease and related higher level of immunosuppression. Again, both administrative data and clinical evaluation were strictly concordant on this point (Table 1). By contrast, the number of hospitalizations, even if in a different period, significantly differed between the administrative source and the clinical source of data, with the latter likely underestimating the events. This highlights the possible bias coming from clinical charts, especially when the time of observation is very long (i.e., 8 years), and, on the other hand, it reinforces the validity of an integrated approach by combining administrative databases and clinical evaluation in long-term retrospective observational studies on rare diseases. Also, the lower prevalence which we reported of MPA, which is often an isolated renal vasculitis managed by nephrologists, reinforces this concept.

The total cost of illness in patients suffering from AAV and residents in the province of Udine from 2010 to 2018 was € 1,215,078, corresponding to € 6168 patient-year. It was more than two times higher than that of patients with giant cell arteritis in the same area [27]. This estimation was the first one reported in the literature coming from a European healthcare system. The universal Italian healthcare system follows a tax-funded model similar to the Beveridge type, which is completely different from the insurance-based systems. With that premise, the substantially higher hospital costs recently reported by Ungprasert et al. from USA are not comparable with our results [3]. The main component of the total cost was represented by costs for hospitalization, amounting to € 734,957 (€ 3731 per person-year). Notably, higher costs of illness for ANCA-positive patients were documented for the first time (Fig. 2) as well as for GPA and MPA if compared to EGPA (Fig. 3) (GPA: € 5199 per person-year vs MPA € 4771 per person-year vs EGPA 2329 per person-year, respectively), therefore implying a higher number of patients with limited disease or with a more favourable course of vasculitis in EGPA than in GPA or MPA [1, 28–31]. However, the association between EGPA with chronic asthma in the adult, that is often resistant to many treatments, may explain the higher costs for prescribed medications for EGPA patients. After vasculitis resolution, asthma remains severe in up to 50% of patients and incidence of isolated-asthma and rhinosinus exacerbations remains constantly high. In addition, in EGPA patients with asthma, long-term severe or uncontrolled asthma is associated with baseline pulmonary and ear, nose, and throat manifestations but not with typical vasculitic features [32]. This observation further supports the consideration of EGPA as a separate entity among AAV.

This healthcare burden was expected in FVG considering the becoming into a chronic disease of AAV, thanks to improvements in treatment. Emerging evidence in other studies [10, 33] is the negative effect on patients’ physical and mental quality of life (QoL) of AAV, comparable to other chronic diseases. Current vasculitis disease assessment tools (BVAS v3 and VDI) are only weakly correlated with measures and variations of QoL [34, 35]. Interesting prospects might be offered by AVV-patient-reported outcomes (PRO) questionnaires. The AAV-PRO questionnaire could be useful to assess the patient’s perspective on the burden of disease [36].

## Strengths and limitations

The main strength of this examination is the achievement of largely homogeneous and overlapping results using health administrative databases, including, of note, even the costs estimated for hospital medications for each patient, and smaller clinical charts. Moreover, our prevalence estimation by healthcare administrative databases (8–9 cases per 100 000 individuals) was close to that reported in the literature of around 2–13 cases per 100 000 individuals [37–39], as well as the inpatient prevalence of 23.2 cases per 100 000 admissions was in line to Li et al. [17]. The advantage of using regional informatics healthcare administrative databases is to obtain available information on patients’ outcomes as well as healthcare burden and costs in a long period, possibly minimizing missing data. The latter are not available with smaller clinical databases, which risk being less informative for retrospective observational studies comprising long-term follow-up. Although not in the present study area, validation studies on rheumatic diseases have been published with good confidence in systemic rheumatic diseases [40, 41]. In addition, a similar study performed in US applied the same methods [42].

A limitation of using administrative health databases is the impossibility to correlate the disease assessment tools, patients’ perception of QoL, and work ability status, with recorded events. However, a complementary clinical evaluation would serve to overcome this limit of a depersonalized disease description. Moreover, a preliminary analysis on work ability status in AAV patients from our cohort has been recently reported, demonstrating the importance of such evaluation as complementary to disease activity and damage in AAV [43]. Also, our work did not allow us to attribute a different impact on costs to different treatment regimens. Probably, more information than that available from retrospective cohorts based on administrative databases would be required to assess the impact of different treatment strategies employed on cost of illness. Controlled studies could more properly address this issue, by avoiding possible confounders. In addition, even if we have access to ED records, they do not include any cost information, as stated in the methods, and thus, our analysis did not consider the economic burden related to ED admission.

## Conclusion

Even if rare, AAV are diseases showing a high level of economic source consumption for the healthcare system. The disease itself and the infectious complications during treatment still remain the main causes of hospitalization and related costs. The ANCA status seems to influence the outcome and the costs of illness greatly. EGPA seems to be a separate entity, also for its healthcare cost profile. Overall, the integration between healthcare administrative databases and clinical charts may draw the best portrait of these rare diseases, when considering a long time of observation.

## Data Availability

The data that support the findings of this study are available on request from the corresponding author, [LQ].
